# Near-Infrared Spectroscopy for Objectifying Cerebral Effects of Laser Acupuncture in Term and Preterm Neonates

**DOI:** 10.1155/2013/346852

**Published:** 2013-05-15

**Authors:** Wolfgang Raith, Gerhard Pichler, Iris Sapetschnig, Alexander Avian, Constanze Sommer, Nariae Baik, Martin Koestenberger, Georg M. Schmölzer, Berndt Urlesberger

**Affiliations:** ^1^Division of Neonatology, Department of Paediatrics, Medical University of Graz, Auenbruggerplatz 30, 8036 Graz, Austria; ^2^Research Group for Paediatric Traditional Chinese Medicine, TCM Research Centre Graz (Acupuncture Research), Medical University of Graz, Auenbruggerplatz 30, 8036 Graz, Austria; ^3^Institute for Medical Informatics, Statistics and Documentation, Medical University of Graz, Auenbruggerplatz 2, 8036 Graz, Austria; ^4^Division of Pediatric Cardiology, Department of Pediatrics, Medical University of Graz, Auenbruggerplatz 34/2, 8036 Graz, Austria; ^5^Department of Pediatrics, University of Alberta, Edmonton, Alberta, Canada T5H3V9; ^6^Neonatal Research Unit, Royal Alexandra Hospital, 10240 Kingsway Avenue NW, Edmonton, Alberta, Canada T5H3V9

## Abstract

Laser acupuncture (LA) becomes more and more relevant in neonates and infants. With near-infrared spectroscopy (NIRS), a continuous and noninvasive measurement of tissue oxygenation is possible. Aim was to investigate, whether the application of LA was associated with any changes in regional cerebral oxygen saturation (rcSO_2_) in term and preterm neonates. The study included 20 neonates (12 males, 8 females). The Large Intestine 4 acupuncture point (LI 4, *Hegu*) was stimulated by a microlaser needle (10 mW, 685 nm laser needle EG GmbH, Germany) for 5 minutes, bilaterally. All neonates underwent polygraphic recording during undisturbed daytime sleep, including heart rate (HR), peripheral oxygen saturation (SpO_2_), and measurement of nasal flow. Using NIRS, rcSO_2_ was measured continuously. Cerebral fractional tissue oxygen extraction (cFTOE) was calculated. We did not observe any significant changes in SpO_2_ and HR values during the whole observation period. However, there was a significant decrease in rcSO_2_ (*P* = 0.003) within postintervention period, accompanied by a significant increase in cFTOE (*P* = 0.010) in postintervention period.

## 1. Introduction

There is an increasing interest in complementary and alternative medicine (CAM) in particular in herbal medicine, homeopathy, and traditional Chinese medicine (TCM) to treat the pediatric population [1–3]. TCM has been practiced in China for over 2000 years as the main form of medical treatment before the introduction of western medicine approximately 100 years ago. TCM includes Tuina (i.e., Chinese massage at acupoints), moxibustion, and all types of acupuncture (acupressure, needle acupuncture, electric acupuncture, and laser acupuncture (LA)) [4]. Positive effects of acupuncture on reduction of pain and agitation in children have been reported [5]. However it is unknown whether repeated needle stimulation may alter sensory processing and responses to subsequent painful stimuli [6] or demonstrates an increased infection risk in premature babies [7]. LA application is painless and can avoid infections, which could be an important alternative to manual acupuncture in infants [8–14]. However, the applied doses, duration of stimulation, peripheral, and central effects of LA are an ongoing discussion [15, 16]. Evidence derived from functional magnetic resonance imaging demonstrated that stimulation of different acupuncture points, for example, LI 4 (*Hegu*) or Liv 3 (*Taichong*) induced specific patterns of brain activity in adults [17] and children [18]. This brain pattern activation is based on the indirect representation of neuronal activity and metabolic changes, particularly the relative changes in concentration of deoxygenated haemoglobin (HHb). Near-infrared spectroscopy (NIRS) has been used to measure cerebral tissue oxygenation and changes in oxygen delivery and oxygen consumption within a tissue compartment [19]. In 1978, Chen and Erdmann [20] firstly described the effects of acupuncture on oxygenation, followed by several studies demonstrating the effect of acupuncture using NIRS in adults [21] and concluded that changes in peripheral and cerebral activities can be quantified and are reproducible using NIRS. The limited evidence of LA studies in children shows similar results compared to adults [22, 23]. However, the central effect of LA in newborn infants has not been evaluated. The aim of the study was to measure changes of regional cerebral tissue oxygenation in term and preterm neonates undergoing LA.

## 2. Materials and Methods

### 2.1. Participants

Preterm and term newborns admitted to the Neonatal Intensive Care Unit of the University Hospital of Graz scheduled for sleep studies were included. Infants were excluded with known genetic anomalies or major malformations, pathology in cranial ultrasound or abnormal neurological examination, elevated bilirubin levels, suspected intrauterine infection, and suspected sepsis or septic shock. Infants >28 weeks gestation were also excluded if they had any need for respiratory support or oxygen during study phase. Infants <28 weeks gestation were only included if they required <30% oxygen or had only needed continuous positive airway pressure. The university ethics committee approved the study, and parental written informed consent was obtained.

Measurements were performed after 2 hours of undisturbed daytime sleep in supine position using a Babytherm 8000 incubator (Dräger GmbH Lübeck, Germany). During the measurements no medication was administered; the ambient temperature and humidity were kept constant. The infant was comforted with a pacifier if needed, and the eyes were protected with an eye shield (Biliband, Natus Medical Inc., San Carlos, CA, USA) as previously described [24].

Heart rate, oxygen saturation, and breathing movements were recorded. Cerebral oxygenation was measured using NIRO 300 (Hamamatsu, Japan). The optodes were placed on the left side of the forehead with an interoptode distance of 4 cm and a sampling rate of 2/s. Light shielding was performed with a slim cap. 

The NIRO 300 continuously measures changes in the oxyhemoglobin (O_2_Hb), deoxyhemoglobin (HHb) concentration, and regional oxygen saturation (rSO_2_ (the NIRO 3oo displays the regional oxygen saturation as “tissue oxygen index (TOI)”). Measurement of rSO_2_ was performed using the Spatially Resolved Spectroscopy (SRS) method, in which the tissue absorption coefficient is determined from the regionally dependent weakening of light. A detector especially developed for the SRS method registers the emitted near-infrared light from the light source into tissue and allowed rSO_2_ calculation using the SRS algorithm. All parameters were stored within a multichannel system.

**Figure 1:**
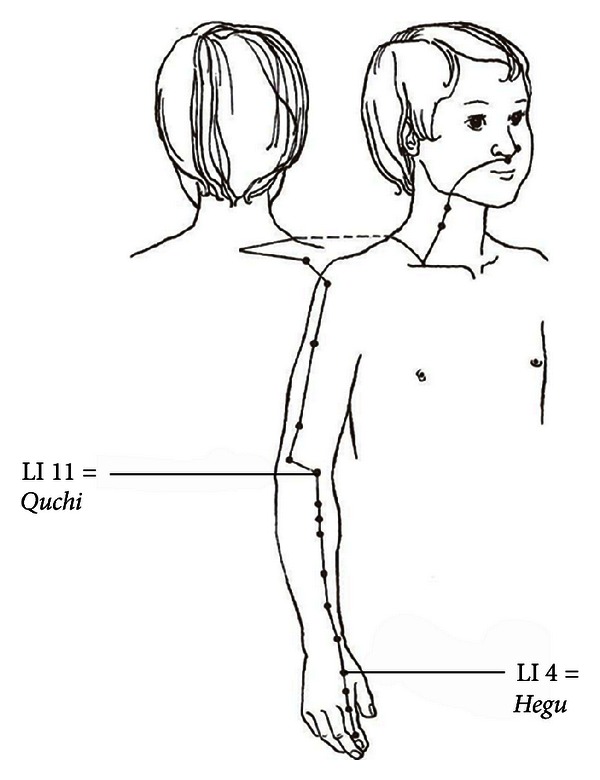
LI 4 (*Hegu*) is located in the Large Intestine Meridian in the middle of the 2nd metacarpal bone on the radial side. The Large Intestine Meridian begins on the index finger and travels along the arm over the shoulder to end outside of the nose. LI 4 (*Hegu*) is considered to be one of the most effective acupuncture points for general pain control, especially of the head. Modified by the author H. Tenk: Internship of Pediatric Chinese Acupuncture, 3 ed, W. Maudrich, 1994.

As acupuncture point Large Intestine 4 (LI 4, *Hegu*) was selected. LI 4 (*Hegu*) is located in the Large Intestine Meridian in the middle of the 2nd metacarpal bone on the radial side (Figure 1). There are a total of 20 points on the Large Intestine Meridian. The pathway begins at the index finger along the arm over the shoulder and ends on the face just lateral of the nose. For LA, a laser needle (laser needle GmbH, Glienicke/Nordbahn, Germany) was used, which has been previously described by Litscher et al. [25–27]. 

The laser needle used for acupuncture provides continuous laser light with a wavelength of 685 nm and an output power of 10 mW per laser needle. An output power of 10 mW (diameter 500 *μ*m) and a radiation time of 5 min resulted in an power density of about 1,5 J/cm² per acupuncture point [27].

Before LA was performed, the skin at the acupuncture point was disinfected, and the laser needles were fixed to the skin with a special adhesive tape, bilaterally at LI 4 (*Hegu*). Once the baby fell asleep, the fixed laser needle was activated for 5 minutes and left undisturbed on the child's LI 4 (*Hegu*), bilaterally (for at least 10 minutes). 

**Figure 2:**
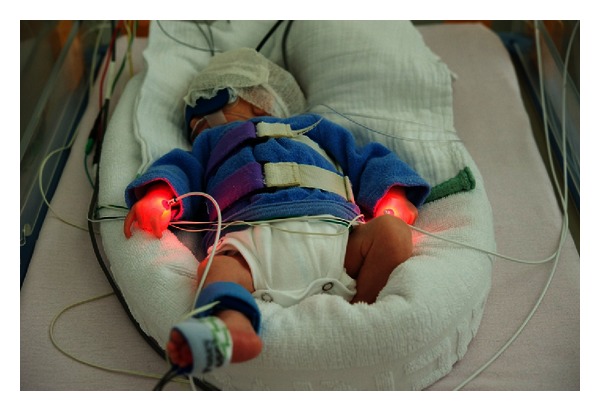
Demonstrates the infant with the applied laser needle and the near-infrared spectroscopy monitoring secured through the slim cap.

Figure 2 shows the infant with the applied and activated laser needle.

To include data in the analysis, there had to be, a “stable period” lasting 3 min before activating the laser needle without body movements, without apnoea or periodic breathing, without variations in heart rate exceeding 15%, and without variations in oxygen saturation measurements. For further analysis, NIRS parameters were recorded before, during, and after laser needle acupuncture with a sampling rate 2/sec.

The prospective protocol consisted of two baseline periods, each lasting 5 minutes, one before (preintervention period), one after intervention (postintervention period). To depict dynamic changes during intervention, the intervention period was divided into 10 periods, each lasting 30 seconds. Mean values of peripheral oxygen saturation (SpO_2_), heart rate (HR), regional cerebral oxygen saturation (rcSO_2_) were calculated for the two baseline periods, as well as for the 10 intervention periods. Cerebral fractional tissue oxygen extraction (cFTOE) was calculated for each period ((SpO_2_ − rcSO_2_)/SpO_2_) [28]. Data are presented as mean and 95% confidence interval. In this analysis, we investigated the changes in SpO_2_, HR, rcSO_2_, and cFTOE within the intervention period compared to preintervention and postintervention periods using a linear mixed model with a fixed effect for time and a first order autoregressive covariance structure. A *P* value of < 0.05 was considered as a statistical significance. The statistical analyses were performed using IBM SPSS Statistics (release 19.0.0. 2010, Chicago, IL, USA, SPSS Inc., an IBM company).

## 3. Results and Discussion

### 3.1. Results

The study group encompassed 20 neonates with a gestational age ranging from 26^6/7^ to 40^6/7^ weeks and a birth weight of 690 to 3680 g. Mean (range) measurements were performed on day 22 (11–68) after birth.  Table 1 shows the demographic data of the study population.

**Figure 3:**
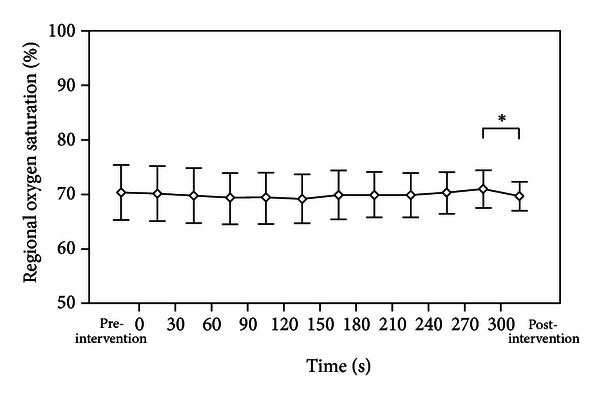
There was a significant decrease in regional cerebral oxygen saturation (rcSO_2_) during postintervention period (*P* = 0.003).

**Figure 4:**
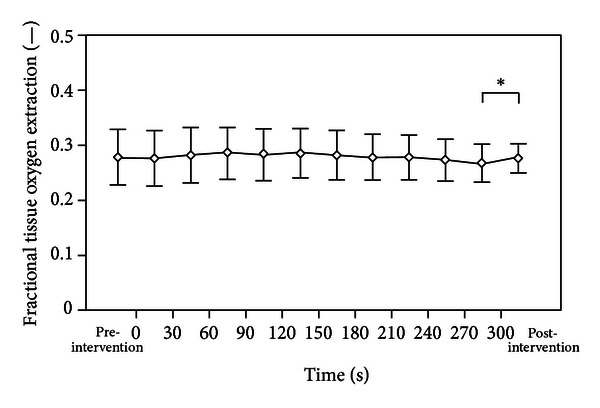
There was a significant increase in the cerebral fractional tissue oxygen extraction (cFTOE) during postintervention period (*P* = 0.010).

We did not observe any significant changes in SpO_2_ and HR values during the whole observation period. However, there was a significant decrease in rcSO_2_ (*P* = 0.003) within postintervention period, accompanied by a significant increase in cFTOE (*P* = 0.010) in postintervention period (Figures 3 and 4).

### 3.2. Discussion

There is an increasing interest in complementary medical treatment of infants and newborn; however, the evidence is scare. A recent meta-analysis demonstrated that [29] acupuncture could be a safe nonpharmacologic treatment option for pain reduction in term and preterm infants. The current study investigated cerebral oxygenation and physiological parameter during LA. The results of the study can be summarised as follows: (i) significant decrease in regional cerebral oxygen saturation (rcSO_2_) in postintervention period, (ii) significant increase in cerebral fractional tissue oxygen extraction (cFTOE), and (iii) no changes at all in peripheral oxygen saturation or heart rate during the whole observation period. This is the first study to demonstrate changes in cerebral tissue oxygenation in association with LA in term and preterm infants. 

Oxygen delivery is the product of blood flow and oxygen content. In neonates, the cardiac output is dependent on HR. In our study, there were no significant differences in HR, which leads us to assume that there were no differences between both groups as regards to blood flow. The oxygen content of arterial blood supplying the brain equates SpO_2_, which did not show any significant changes either. Therefore, we hypothesize that the observed significant changes in rcSO_2_ and cFTOE were due to changes in regional cerebral tissue perfusion and oxygen extraction. Our findings suggest that there was a decrease in local arterial blood supply after discontinuation of LA, expressed in a decrease of rcSO_2_. This was accompanied by an increase in regional tissue oxygen extraction, expressed in an increase of cFTOE. Furthermore, we hypothesize that the increase of local cerebral tissue perfusion may have been a slow process, and that may be the reason why there were no significant changes measureable during LA. There are some similarities to reported results of LA in adults [27, 30]. Litscher and Wang reported an increase of O_2_Hb and TOI after needle and laser acupuncture in adult patients [31]. Furthermore, Litscher described a possible correlation between manual needle acupuncture, laser acupuncture, electrical punctual stimulation, and changes in regional cerebral oxygenation in more than 100 volunteers [32]. 

LI 4 *(Hegu*), which is considered to be one of the most effective acupuncture points for general pain control, was used in the current study. Manual acupuncture applied to LI 4 (*Hegu*) activates the sympathetic and parasympathetic nervous systems in healthy individuals [33]. Stimulating LI 4 (*Hegu*) bilaterally resulted in a more immediate effect than unilateral stimulation [34]. LI 4 (*Hegu*) has been used to treat infantile colic [35–38] and has been described for analgesia [39]. Furthermore, LI 4 (*Hegu*) induces specific patterns of brain activity in adults and children during manual acupuncture, electroacupuncture, and LA [40, 41]. These investigations are based on the indirect representation of neuronal activity and the resulting metabolic changes, particularly the relative changes in concentration of HHb. NIRS is an established approach to noninvasive measure peripheral and cerebral tissue oxygenation [42]. Near-infrared light penetrates deep into the tissue allowing to monitor tissue oxygenation. The oxygen-dependent absorption of light by haemoglobin enables the calculation of relative changes in the oxygenated and deoxygenated haemoglobin [43]. The advantages of NIRS are (i) noninvasive, (ii) low risk, (iii) continuity, and (iv) particularly suitable for the neonatal population due to their thin scalp and skull. The application of the method is easy, and it is used in several studies to measure cerebral [44–46] and peripheral oxygenation in term and preterm newborn [47, 48]. Our results demonstrate that NIRS can be used to measure changes of cerebral tissue oxygenation in term and preterm neonates undergoing LA.

Currently there are only a few studies that investigated the effect of acupuncture in neonates [49]. In children, acupuncture has been demonstrated to have positive effects on pain [39, 50, 51]. In comparison, there is a lack of data in newborn infants about safety of acupuncture and the response to acupuncture. Current evidence suggests that acupuncture is a safe modality for pediatric patients. However, fewer needles should be used when treating infants compared to adults [52, 53]. Case reports and case series have been described for neonates, and early infancy has been carried out, for example, as a therapy for infantile colic [35–38, 54], pain treatment, and newborn abstinence syndrome [55, 56]. A major limitation of acupuncture in newborns is their skin vulnerability with a potential to damage the skin resulting in a potential entry wound for infectious diseases. It is also unknown whether repeated needle stimulation may alter sensory processing and responses to subsequent painful stimuli, in the same manner like heel sticks, necessary to take blood samples [6, 39, 57]. LA is a painless procedure, and therefore, it becomes a more and more relevant alternative to manual acupuncture in infants [7–14]. But the central and peripheral effects and the applied doses in neonates and infants undergoing laser acupuncture are a matter of fact in ongoing discussions. Recently, acupuncture was considered to be included in the pain management algorithm for children as an effective nonpharmacological approach [58, 59].

The current study has some additional limitation not previously mentioned. The acupuncture effect of laser stimulation depends on the power density at the acupuncture point. 

For a laser output power of 10 mW, the resulting power density at the acupuncture point is in the order of 5 W/cm². An output power of 10 mW and a time of radiation of 5 min result in an energy dosage of about 1,5 J/cm². Maybe with a higher energy dosage (higher laser output power and/or a longer radiation time) the results could be more significant, especially during LA [27]. 

But based on our recent published data about the changes of the skin temperature in preterm infants undergoing laser acupuncture [15], it seems rational and safe to use the same laser needle with the same output power (10 mW) and the same time of stimulation (five minutes). Another limitation of our study was the small number of infants included in the study. Future research should focus on alternative or adjunctive nonpharmacological therapy to understand the utility, safety, and effectiveness of acupuncture in newborns and infants and investigate central effects of LA in neonates, by changing the time of stimulation and/or the energy doses.

## 4. Conclusions

There was a significant decrease in rcSO_2_ during postintervention period. This was accompanied by a significant increase in cFTOE. This was in contrast to SpO_2_ and HR, where no changes could be observed. Therefore, we hypothesize that observed changes were due to changes in regional cerebral perfusion and oxygen supply. This is the first study to demonstrate changes of cerebral tissue oxygenation caused by laser acupuncture in term and preterm neonates.

## Figures and Tables

**Table 1:** Values are median and range for continuous data and absolute counts for categorical data.

Investigated neonates	*N* = 20
Male/female	12/8
Preterm/term	17/3
Birthweight	2120 g (690 g–3600 g)
AGA/SGA	19/1
GA (in completed weeks)	34 + 0 (27–40)
Day of life at the time of investigation	22 (11–68)
GA (in completed weeks) at the time of investigation	37 + 2 (34–42)
Weight at the time of investigation	2353 g (1882–3685 g)
HCT in %	43.4%

GA: gestational age; AGA: appropriate for date; SGA: small for date; HCT: hematocrit.

## Abbreviations

CAM: Complementary and alternative medicine
TCM: Traditional Chinese medicine
LA: Laser acupuncture
NIRS: Near-infrared spectroscopy
O_2_Hb: Oxyhemoglobin
HHb: Deoxyhemoglobin
TOI: Tissue oxygenation index
HR: Heart rate
SpO_2_: Peripheral oxygen saturation
rSO_2_: Regional oxygen saturation
rcSO_2_: Regional cerebral oxygen saturation
cFTOE: Cerebral fractional tissue oxygenation
SRS: Spatial resolved spectroscopy
LI 4 (*Hegu*): Large Intestine 4.
